# Migrant women’s experiences, meanings and ways of dealing with postnatal depression: A meta-ethnographic study

**DOI:** 10.1371/journal.pone.0172385

**Published:** 2017-03-15

**Authors:** Virginia Schmied, Emma Black, Norell Naidoo, Hannah G. Dahlen, Pranee Liamputtong

**Affiliations:** 1 School of Nursing and Midwifery, Western Sydney University, Sydney, New South Wales, Australia; 2 Perinatal and Women’s Mental Health Unit, St John of God Health Care and School of Psychiatry, University of New South Wales, Burwood, New South Wales, Australia; 3 Ingham Institute for health and Medical research, Liverpool, New South Wales, Australia; 4 School of Science and Health, Western Sydney University, Sydney, New South Wales, Australia; University of Ottawa, CANADA

## Abstract

**Aim:**

To conduct a meta-ethnographic study of the experiences, meanings and ways of ‘dealing with’ symptoms or a diagnosis of postnatal depression amongst migrant women living in high income countries.

**Background:**

Prevalence of postnatal depression is highest amongst women who are migrants. Yet many women do not seek help for their symptoms and health services do not always respond appropriately to migrant women’s needs. Studies have reported migrant women’s experiences of postnatal depression and it is timely to synthesise findings from these studies to understand how services can be improved.

**Design:**

A meta-ethnographic synthesis of 12 studies reported in 15 papers

**Data sources:**

Five databases were searched for papers published between January 1999 and February 2016

**Review methods:**

The quality of included studies was assessed using the Critical Appraisal Skills Program tool. The synthesis process was guided by the seven steps of meta-ethnography outlined by Noblit and Hare.

**Findings:**

Four key metaphors were identified: “I am alone, worried and angry—this is not me!”; ‘Making sense of my feelings’ ‘Dealing with my feelings’ and ‘What I need to change the way I feel!’. Primarily women related their feelings to their position as a migrant and as women, often living in poor socio-economic circumstances and they were exhausted keeping up with expected commitments. Many women were resourceful, drawing on their personal strengths and family / community resources. All the studies reported that women experienced difficulties in accessing appropriate services.

**Conclusion:**

The meta-ethnographic study demonstrates the impact of migration on perinatal mental health, particularly for women lacking family support, who have no employment, a precarious migration status and/or relationship conflict. Migrant women are resourceful and this requires support through appropriate services. Further research is needed to evaluate effective support strategies for migrant women in the perinatal period.

## Introduction

Poor mental health, particularly depression and anxiety in pregnancy and the year after birth, is a global public health issue, associated with poorer outcomes for women and their infants [1–4]. Epidemiological studies demonstrate poorer cognitive, language and physical development as well as psychosocial, emotional and behavioural problems in children whose mothers experienced pre and or postnatal depression (PND) and anxiety [1]. Prevalence rates of PND reported in meta-analyses and systematic reviews range widely, from 2% to 82% in high income countries (HIC) and from 5% to 74% in low and middle income countries (LMIC) (using self-report) [5]. By contrast, when determined by clinical interview, prevalence of PND remains variable but lower, from 0.1% in Finland to 26.3% in India, respectively [5]. However, it is concerning that rates of postnatal depression for women who are migrants appear to be considerably higher than the non-migrant populations [6–8]. A recent systematic review and meta-analysis suggests that about 20% of migrant women experience PND symptoms in the first year following childbirth and this is one and a half to twice as likely when compared with non-migrant women [7]. Risk factors for PND in migrant women included a shorter length of residence in the receiving country, lower levels of social support, poorer marital adjustment, and perceived insufficient household income [7]. Notably the studies in this review were all conducted in HIC.

Studies report reluctance by women, particularly migrant women to take up services for postnatal depression [9, 10]. Cultural beliefs and language barriers impede women’s help-seeking [11–17]. Studies also reveal that health services are not always accessible or culturally appropriate to meet the needs of migrant women [17].

Given the higher prevalence of depression amongst migrant women and the apparent reluctance to seek help, it is important to understand women's experience of this phenomenon so that appropriate services can be offered. A number of researchers have used qualitative methods to explore women's experiences of PND. A 2002 metasynthesis of 18 qualitative studies [18] identified four themes, ‘incongruity between expectations and the reality of motherhood’, ‘spiralling downward’, ‘pervasive loss’ and ‘making gains’. [18]. Knudson-Martin and Silerverstein [19] used grounded theory techniques to reanalyse reported data in nine qualitative studies, concluding that PND must be considered as a relational concern and not simply bio-medical illness. Most recently, a meta-synthesis of 12 studies identified four themes which reflect the progression of PPD—‘crushed maternal role expectation’, ‘going into hiding’, ‘loss of sense of self’, and ‘intense feelings of vulnerability’, all of which they argued were exacerbated by practical life concerns such as financial issues [20]. While migrant women have participated in some of the studies included in these meta-syntheses, their specific needs and concerns and the impact of cultural factors upon PND was not addressed.

Two recent integrative reviews [21, 22] identified the increased prevalence, the differential risk factors for PND and the barriers to help seeking amongst migrant women. Both reviews concluded that a broad range of system responses is required, from the macro- (e.g. equitable immigration policies) to the individual level (e.g. addressing cultural barriers to help-seeking) [21, 22]. O’Mahoney et al [22] emphasised that more research was needed to hear migrant women’s ideas about their social support needs, the difficulties they experience and their preferred ways of getting help with PPD.

The aim of this paper is to report the findings of a meta-ethnographic study of the experiences, meanings and ways of ‘dealing with’ symptoms or a diagnosis of postnatal depression amongst migrant women living in high income countries with a view to informing culturally appropriate health service design and delivery.

## Method

The synthesis of studies presented in this paper was guided by the original work of Noblit and Hare [23] on meta-ethnography. They described seven phases in their approach: [1] ‘getting started’–deciding the focus of the synthesis; [2] ‘deciding what is relevant to the initial interest’—selecting studies to synthesise; [3]; ‘reading the studies’ repeatedly and noting of metaphors, concepts, themes; [4] ‘determining how studies are related’ by juxtaposing concepts/metaphors from studies to see how they relate to each other; [5] ‘translating the studies into one another’ by comparing concepts/metaphors between and within accounts; [6] ‘synthesising translations’ by seeing if there are common types of translations or if some translations or concepts can encompass those from other studies; and [7] ‘expressing the synthesis’—that is communicating it to the identified audience. While Noblit and Hare outlined these processes to synthesise ethnographic studies, others have used these techniques when synthesising studies that have adopted a range of qualitative methodologies in diverse healthcare settings [24–28]. The synthesising of qualitative findings is more than simply summing up or reducing the findings to one common conclusion; rather, this approach offers the opportunity to enlarge the interpretive possibilities of findings [29, 30].

### Search strategy

The literature search was designed in collaboration with a research librarian at the university and was conducted between September and December 2015 using the following databases: CINAHL, MEDLINE, EMBASE, PsycInfo and SCOPUS. An additional brief search was conducted in Scopus in late March 2016 to identify any new citations of the papers already identified for inclusion. No new papers were identified at this stage. Key concepts were mapped to search terms (including EMTREE and MESH terms) (summarised in Table 1). Relevant terms such as ‘migrant’, ‘pregnancy’, ‘postnatal depression’, were also included as key words.

**Table 1:** Search terms.

Migrant	AND	Pregnancy	AND	Depression
Migrant*, Refugee*, Immigrant,* Immigration, Transient,* Emigrant*, Cross cultural differences (PsycInfo)		Pregnan*, Perinatal*, antenatal, postnatal*, postpartum*, pregnancy outcomes (PsycInfo); Mothers (PsychInfo)		Depression, puerperal, depression, antenatal depression, postnatal depression, postpartum depression, perinatal depression, perinatal mental health

To be included in the synthesis, participating women had to be a first generation migrant and have reported symptoms of, or had been diagnosed as having, PND. The term migrant is defined by the International Organization for Migration (IOM) as “any person who is moving or has moved across an international border or within a State away from his/her habitual place of residence, regardless of 1) the person’s legal status; 2) whether the movement is voluntary or involuntary; 3) what the causes for the movement are; or 4) what the length of the stay” [31]. This broad definition therefore includes social and economic migrants, humanitarian migrants, as well as those seeking asylum. Papers that included both migrant women (first generation) and those born in the host country (second generation) were retained provided that the authors had identified the source of their data reported and that the data from recent migrants had contributed to building the themes that arose in the individual studies. In the search strategy we did not limit the country of origin or the receiving country by the use of terms such as low and middle income country (LMIC), high income country (HIC). However, in selecting the studies for the review we only included studies where women had migrated to HIC. This was for three reasons; 1) the majority of migrants live in HIC [31], 2) most research is conducted in HIC and 3) our goal was to use the findings of the study to inform policy service responses in HIC such as Australia and countries with similar level of access to maternity and specialist perinatal mental health services but where there continues to be an underutilisation of mental health services particularly by migrant women who were born in a non-English speaking country [32]. Papers reporting only on health professionals’ perspectives of the experiences of migrant women with PND were excluded.

Studies were limited to those published in English, peer-reviewed journals between January 1999 and March 2016. This period was selected as the earliest qualitative studies to examine migrant women’s experiences of PND were published in 1999 [33, 34]. Meta-ethnographic syntheses rely on rich qualitative data primarily generated through qualitative studies. We did include mixed-methods’ studies if sufficient qualitative data were reported. Only one mixed methods study was included [35]. We also considered including case studies or individual narratives but did not locate these in our search

### Data quality

Quality appraisal was undertaken by three authors (VS, EB, NN) using the Critical Appraisal Skills Programme (CASP) tool for qualitative research [36]. All papers assessed for inclusion were required to meet the two essential criteria outlined in the CASP tool: clarity of study aims and appropriateness of methodology (a qualitative approach). The remaining eight CASP questions cover different aspects of research such as ethics, clarity of design, recruitment, data collection methods and analysis. We determined that any papers that scored six out of 10 or above would be included in the review.

### Data extraction and synthesis

The first step in data extraction was to tabulate key descriptive data from the studies including country, study aim, participants (including age range, country of birth, length of time in receiving country), study methodology and methods (including if the participants were interviewed in their first language) and key findings. Data extraction and synthesis was guided by phases four, five and six used in meta-ethnography articulated by Noblit and Hare [23]. Noblit and Hare [23] described three techniques for translation; 1) reciprocal translation (looking for similarities across studies); 2) refutational investigation (identifying differences or challenges to the emerging concepts) and 3) the development of a ‘line of argument’ that takes into account both the similarities and differences found in the studies [29]. In some instances authors may use all three approaches but it is much more common that reciprocal translation is the main or only process used [29]. In this study we used the technique of reciprocal translation because the similarities across the studies dominated. Noblit and Hare suggest that translation involves examining the key concepts in relation to others in the original study and across studies. The way of translating key concepts or interpretive metaphors from one study to another involves an idiomatic rather than a word-for-word translation. Three authors (VS, NN, EB) read each paper and identified the common themes with data exemplars. Themes and subthemes from papers were all extracted into a word document with data exemplars and key ideas from authors presented in results or discussion. Pre-existing themes which were more abstract or conceptual such as “being a good mother” and “I just get on with it” were retained and new themes and sub theme developed as necessary. All authors then reviewed and confirmed the final themes and subthemes. The studies included in the meta-ethnography are listed in Table 2.

**Table 2:** Papers include in the meta-ethnographic study.

Author, year, country	Aim	Participants	Methodology & Methods	Findings	CASP score
Ahmed, A. Stewart, D. E., Teng, L., Wahoush, O., Gagnon, A. J., (2008)[42] Canada.	To understand experiences and attributions of symptoms; experiences with services; factors that facilitated or hindered help seeking and recovery.	10 participants with an EPDS score of ≥10. Women were born in China (n = 2), India (n = 2), Pakistan (n = 1), South America (n = 3), Egypt (n = 1) and Haiti (n = 1) and had lived in Canada < five years.	A ‘qualitative study’ using semi-structured telephone interviews. Seven women were interviewed in English and interviews were conducted in languages other than English (French, Mandarin, Spanish) and later translated into and transcribed in English (do not report by whom). Data analysed using the constant comparative method.	Five Themes: 1) Experience and attributions of causes of depression, 2) Experience with services, 3) Barriers to asking for help, 4) Attributions of causes of recovery, 5) Difference between women still sad and depressed and women who had recovered. Contributed to Themes 1,2,3 and 4	8/10
Callister, L., Beckstrand, R, Litt, E., Souza, G., Corbett, C. (2010)[11] USA	To describe perceptions of immigrant Hispanic women experiencing symptoms of PND; to identify barriers to help-seeking.	20 immigrant Hispanic women aged 17 to 39 years (mean = 24) (19 from Mexico & one from Argentina) scoring positive for symptoms of PND determined by using the Postpartum Depression Screening Scale Spanish Version. Details on immigrant status collected but not reported.	A qualitative descriptive study. Semi-structured interviews conducted in Spanish by a multilingual and bicultural member of research team. This researcher transcribed and translated the interviews.	Three Themes: 1) Personal Barriers 2) Social Barriers 3) Health Care Delivery Barriers	8/9
Gardner, P. L., Bunton, P., Edge, D., Wittkowski, A. (2014)[40], UK	To improve understanding of West African women's experiences of PND.	Six immigrant women (three were born in Nigeria and three were born in Ghana, length of time in UK not described. Each woman had scored >nine on EPDS.	Interpretive Phenomenological Approach. Semi-structured interviews, all conducted in English (inclusion criteria—fluent in English). Data analysed using	Five themes: 1) conceptualising PND; 2) isolation; 3) loss of identity 4) issues of trust; 5) relationships as a protective factor	7/10
Mamisachvili, L., Ardiles, P., Mancewicz, G. Thompson, S. Rabin, K., Ross, LE. (2013)[41] Canada	To compare the experiences between first- and second generation. Canadian women in order to understand the impact of cultural values, beliefs, and immigration experiences on Post-Partum Mood Problems.	N = 9 First generation mothers average age 33.4 years (n = 5 from Chile, Uruguay, Guatemala, Ethiopia, Rwanda). Length of time in Canada ranged from two to 15 years (mean = 8.3 Years). N = 8 second generation mothers’, average 33.9 years first order data excluded from current metasynthesis	Methodology not specified; however used thematic analysis and semi-structured interviews all conducted in English (inclusion criteria—fluent in English)	Four themes: 1) PPMP stigma- both within context of culture of origin and Canadian culture; 2) relationship with parents/in-laws 3) expectations of managing motherhood 4) identity issues/relationship with self	7/10
Morrow, M., Smith, J. E., Lai, Y. Jaswal, S. (2008)[32] Canada	To examine: 1) women's experiences of PND 2) variables associated with the experience of depression after childbirth, 3) the role of women's family and community 4) support needs.	18 Punjabi-speaking, Cantonese-speaking and Mandarin-speaking immigrant women and one second-generation immigrant woman. Length of time in Canada ranged from approx four to 40 years. Women had either received a diagnosis of PND or self-identified as having experienced depression following child birth.	Ethnographic narrative approach. Semi-structured open-ended interviews. Conducted in the first language of participants by bilingual members of the research team	Three Themes: 1) Women's experiences and expressions of PPD. 2) Psychosocial Stresses—The migration experience, Adherence to gendered roles, the roles of mothers in society and conflicts with family members, The desire for boy babies. 3) The role of family, community and social support and help seeking, 4.The role of health care professionals.	9/10
Nahas, V. L. Amasheh, N. (1999)[33] Australia.	To examine the care meanings and expressions of PND among Jordanian immigrant women in Sydney.	22 Jordanian Australian women. Nine key informants and 13 general informants. No further demographic details provided. Women were diagnosed as suffering from PND (screening method not reported)	Ethnonursing research method (Leininger, 1991). Interviews conducted in either Arabic or English. One of the authors speaks Arabic	Three Themes: 1) Care means carrying and fulfilling traditional gender roles as mother and wife. 2)Care means strong family support and kinship ties during the postpartum period. 3) Care is preservation of the Jordanian childbearing customs as expressed in the celebration of the birth of the baby.	8/10
Nahas, V. L., Hillege, S and Amasheh, N. (1999)[34] Australia	To explore the lived experiences of PND among Middle Eastern women living in Sydney, Australia.	45 Middle eastern women (18 Lebanese, 14 Egyptian, and 13 Palestinian) ranged 19 to 38 years (mean 28.3 years) self-reported experience of PND. Length of time in Australia not clear but had lived in Sydney for the past five years.	Phenomenological research design & analysis used to conduct and analyse in-depth, unstructured interviews. Interviews conducted in either Arabic or English. One of the authors speaks Arabic	Five Themes: 1) Loneliness due to feelings of isolation and lack of social support. 2) Helplessness due to inability to cope with the overwhelming task of fulfilling her traditional role as mother and wife. 3) Fear of failure and being labelled a "bad mother" by in-laws. 4) Insufficient knowledge about postpartum depression and available support services. 5) Coming to terms with PPD by undertaking diversional activities and learning new skills.	8/10
O’Mahony, J. Truong Donnelly, T., Raffin Bouchal, S. & Este, D. 2012a [44] Canada	To understand the factors influencing help-seeking behaviour in immigrant and refugee women, surrounding PPD. To increase understanding of the mental health needs of immigrant and refugee women after childbirth.	30 women described by authors as having immigrant or refugee status and living in Canada for < 10 years (8 were refugees and 22 were immigrants) who had either been formally diagnosed with PND or had an EPDS score of ≥10. Women were from Mexico (n = 8) South America (n = 4), Central America (n = 1), South East Asia (n = 1) South Asia (n = 3), China (n = 5), the Middle East (n = 6) and Africa (n = 2). Years living in Canada– 14<2years; nine women two- to- five years; seven women six to 10 years.	Critical ethnography, Semi-structured, open ended interviews conducted in the language the participant preferred. Professionally trained female interpreters were used in interviews with 12 women. Data were analysed based on Carspecken (1996), Sandelowski (1995) and Denzin and Lincoln (1994). 10 Participants re-interviewed to confirm and further explore interpretations. Varying frameworks applied in the individual papers (see below)	Four themes emerged from data analysis: 1) the conceptualization of PND, 2) complex challenges in seeking help, 3) facilitators in seeking help, 4) intervention strategies for care and treatment.	9/10
O’Mahony, J. Donnelly, TT., Raffin Bouchal, S. & Este, D. 2013[46] Canada	(1) Understand factors that influence immigrant and refugee women’s mental health care experiences (2) Explore service needs and appropriate strategies to address PPD in these populations		Analysed using Kleinman’s explanatory model.	Three Themes: 1) Cultural influence in seeking support 2) Socioeconomic influence in seeking support and 3) Spiritual and religious beliefs.	9/10
O’Mahony, J. Donnelly, TT., Raffin Bouchal, S. & Este, D. 2012b [45] Canada	To understand health service utilisation & social support networks and needs among immigrant and refugee women in relation to PND.		Analysed using Kleinman’s explanatory model.	Two Themes: 1) Formal support given. 2) Informal support.	9/10
O’Mahony, J. & Donnelly, TT. 2013 [43] Canada	The aim of this study was to explore with participants how the roles of gender, race and class might affect the ways in which they access PPD services.		Employed postcolonial feminist perspective to interpret data.	Two Themes: 1) Immigration policy as a barrier 2) Gender role as a barrier to help seeking.	9/10
Ornelas, IJ, Perreira, KM., Beeber, L., Maxwell, L., (2009)[47] USA	To understand factors leading to the development of depressive symptoms among Mexican immigrant mothers	20 low-income, Mexican-born mothers, mean age 27 years. Half (n = 10) scored higher than 16 on the CES–D Scale. The average length of residence in the US was five years, and the average age at arrival in the US was 23 years.	Informed by Family Stress Model Semi structured interviews, conducted in Spanish by a trained bilingual research assistant who had an ongoing relationship with the women as part of the larger study. Thematic analysis	Factors impacting on maternal mental health: 1) Economic stressors 2) Social stressors. Coping strategies: 1) Support from husbands; 2) Support from female friends and relatives; 3) Accessing community resources.	8/10
Parvin, A. Jones, C. E. Hull, S. A. (2004)[38] UK	To understand Bangladeshi women's understandings, experiences and coping strategies used to deal with postnatal distress.	25 Bangladeshi women living in Tower Hamlets, east London. All born in Bangladesh, ranged in age from 21 to 54 years (mean 34.3) and spoke little English. Mean time living in UK 9.97 years	Three focus groups with 10, eight and seven women, respectively. Thematic content analysis used to interpret data. Focus groups conducted in Sylheti, a dialect from northern Bangladesh by one of the researchers.	Four Themes: 1) Experiences of giving birth in the UK 2) Family circumstances after birth 3) Responses to emotional distress and problems within the family. 4) Experiences of primary care services in the postnatal period	7/10
Shafiei, T., Small, R, McLachlan, H. (2015)[35] Australia	To investigate immigrant Afghan women's emotional wellbeing and experiences of PND; to explore health service utilisation in relation to this.	Total sample 39 Afghani women (11< 25years old; 20 between 25 and 34 years and seven >35 years old). 20 had been in Australia <5 years, nine between six to 10 years and 10>10 years, 10 Afghan women participated in in-depth interviews.	Mixed Methods design using a semi-structured telephone interview with closed and some open questions and an in-depth face to face interview. Telephone interviews were conducted four months after the birth in women's preferred language (Dari/Persian or English) by one of the authors, a bilingual researcher Thematic content analysis used to interpret data	Two themes:1) feeling alone and lack of support 2) being overwhelmed. Reasons for not seeking help with emotional wellbeing: 1) reluctance due to discomfort; 2) belief that health professionals wouldn’t be able help 3) belief that health professionals wouldn't want to deal with emotional issues	9/10
Templeton, L. Velleman, R. Persaud, A. Milner, P. (2003)[39], UK	To describe the experiences of women suffering from postnatal depression in black and minority ethnic communities.	Six women participated in interviews; Scored 12 or above on the EPDS. These women were classed as Bangladeshi, Indian, other Asian and ‘other’ (in this latter group there were two women from Portugal and one woman who identified herself as mixed race). 12 additional women participated in focus groups No information on how long women had been in UK	Semi-structured interviews and focus groups. Data analysed using descriptive thematic analysis. Interviews with four women were conducted in English and in two an interpreter was used. All focus groups were conducted in English.	Three Themes: 1) Issues specific to pregnancy and birth (Incl. Postnatal Depression). 2) Issues specific to health care. 3) Issues specific to culture.	7/10

## Results

### Search results

**Fig 1:**
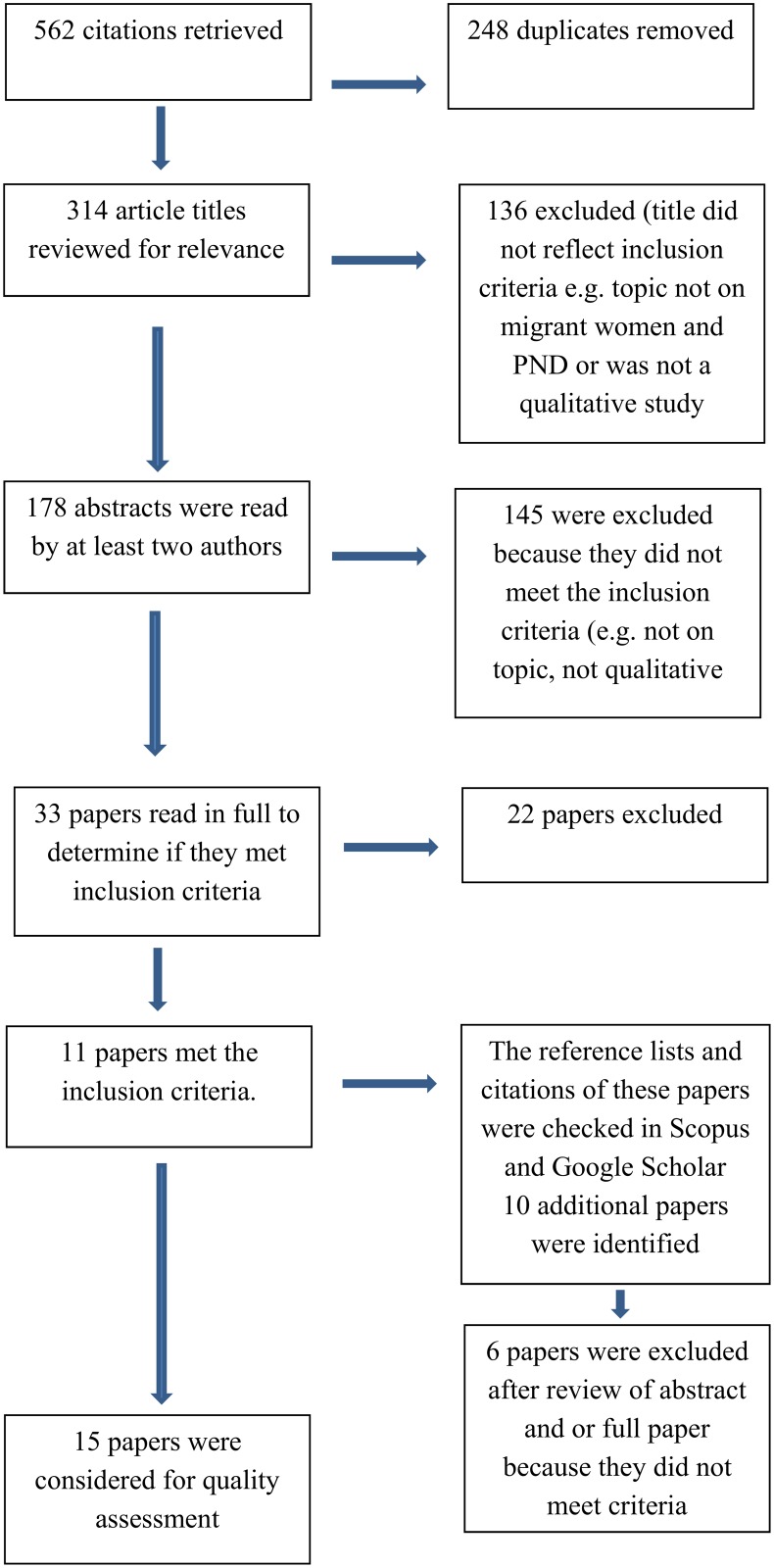
Search results.

The search generated a total of 562 citations. Duplicates were removed (248 citations) leaving 314 papers. The titles of these 314 papers were reviewed by two authors (EB and NN) and 136 were excluded because the title did not reflect the inclusion criteria, primarily either the focus of the paper was not on migrant women and PND or it was clear it was not a qualitative study. The abstracts of 178 papers were read by EB, VS and NN and 145 were excluded at this point as they did not meet the inclusion criteria. Thirty three papers were read in full by the three reviewing authors and 11 met the inclusion criteria. Four of these papers reported different aspects from one study by O’Mahony in Canada. A further 10 papers were found through ‘backtracking’ by searching references lists of the 11 papers [37] and citations of these papers. Four of these met the inclusion criteria [32, 33, 38, 39]. Fifteen papers were then assessed for quality for inclusion in this meta-ethnographic synthesis (see Fig 1).

Consensus on the quality of the 15 papers was reached by the three reviewing authors (VS EB NN). All papers met the first two CASP criteria. Total scores across the papers varied from 7/10 to 9/10 (see Table 2) and therefore all were included in the review. The most common limitations were (a) limited discussion of theoretical or conceptual perspectives and (b) reflection on the roles of the researchers. Given the importance of language in talking about experiences and expressing emotions, it was important to identify whether the interviews were conducted in the participants’ first language by a bilingual researcher or with an interpreter present. or whether authors had specifically excluded participants who did not speak English. In 10 of the 12 studies, women were offered the option to have an interview in their first language and in two studies [40, 41] women who did not speak English were excluded (see Table 2).

The 15 papers reporting findings from 12 individual studies were reviewed and synthesised. A total of 256 women participated in these 12 studies which were conducted in Canada, US, UK and Australia. Participants had migrated from a wide variety of different countries within Central America, the Caribbean; South America; Asia, Africa and the Middle East. The length of time in the receiving country ranged from less than two years to up to 40 years. Most studies did not limit the length of residence and included all migrant women who met the criteria for inclusion. Some were more specific; for example, Ahmed [42] included women living in Canada for less than five years; O’Mahony et al [43–46] included women who had been in Canada less than 10 years; and Nahas, Hillage and Amasheh included Middle Eastern women living in Sydney in the past five years [34]. Some authors did not specific the length of time in the country [11, 33, 39, 40].

The women who participated in these studies all had either been identified as having symptoms of PND or had been diagnosed with depression and were recruited to the studies through intervention and support services. Key metaphors that appeared across studies were: “**I am alone, worried and angry—this is not me!**”; “**Making sense of my feelings”**; ‘**Dealing with my feelings”** and ‘**What I need to change the way I feel!**’ (Table 3 below shows the themes and subthemes).

**Table 3:** Study themes and subthemes.

Author, year, country	I am alone, worried and angry	Making sense of my feelings				Dealing with my feelings			What I need to change the way I feel	
	(Sub themes combined here)	You cannot talk about PND	Life as a migrant is difficult	Loss of social support and cultural traditions	Being a ‘good’ mother and wife is culturally expected	I just get on with it	I am strong	It’s good to talk and feel cared for	I need more information	They don’t understand me
Ahmed et al (2008)	X	**X**	**X**	**X**			**X**	**X**	**X**	**X**
Callister, et al (2010)	x				**X**			**X**	**X**	**X**
Gardner et al (2014)	x	**X**	**X**	**X**				**X**	**X**	**X**
Mamisachvili et al (2013)	x				**X**		**X**		**X**	**X**
Morrow et al. (2008)	x	**X**	**X**		**X**	**X**	**X**	**X**	**X**	**X**
Nahas & Amasheh (1999)	x	**X**		**X**	**X**		**X**			**X**
Nahas Hillege, (1999)	x	**X**		**X**	**X**				**X**	**X**
O’Mahony et al (2012a)		**X**	**X**	**X**			**X**	**X**	**X**	**X**
O’Mahony et al (2013)		**X**			**X**		**X**			
O’Mahony, et al (2012b)				**X**				**X**	**X**	**X**
O’Mahony, J. & Donnelly, TT. (2013)			**X**	**X**	**X**			**X**	**X**	**X**
Ornelas, et al (2009)	x		**X**	**X**				**X**	**X**	**X**
Parvin, Jones, Hull, (2004)	x	**X**		**X**		**X**	**X**		**X**	**X**
Shafiei, Small, McLachlan, (2015)	x		**X**	**X**		**X**	**X**		**X**	**X**
Templeton, et al (2003)	x	**X**		**X**	**X**			**X**	**X**	**X**

### Theme 1: I am alone, worried and angry—This is not me

Overwhelmingly the studies revealed that women experienced a deep sense of loneliness, they were worried about themselves and their children and some felt anger at their situation. This theme also includes several sub themes demonstrating the loss of sense of self; a feeling of desperation and concern that others would think she was crazy.

The loneliness and isolation [42] was described by one participant as “*I have nobody*, *it’s just like you are an island on your own*. *I have got nobody to help me*’ ([40], p.759). Women described feeling irritable, exhausted, becoming over-emotional and crying easily [39, 40, 42]: “*I snapped all the time*, *unnecessary things would just get to me and I would just fly up*” [40]. Some women felt angry and concern that their anger was directed at their children.

I began to feel very angry. I would get angry and wanted my children to get away from me and didn’t want anyone to talk to me. I wanted to lay in my room alone.

([32], p.607)

Women also worried constantly about many aspects of their lives, particularly their baby and family, “*I felt lonely*, *I felt like it's scary to have these two kids to be looked after all by myself and do everything for them*” ([35], p.674). This worry made some unsure of their mental state: “*It’s like your head/mind becomes crazy*, *because I worry all the time”(*[38], p.256).

Many women expressed their feelings through metaphors. In one study, Mandarin-speaking women used analogies to describe emotions, for example: “*Like a rising stream of water I couldn’t find any outlet”… “like threads all tangled up”…“It felt as if the sky was going to fall down on me*” ([32], p.602). Bangladeshi women described an aching, trembling or sensation of pressure in their hearts ([38], p.256).

#### I am not myself

A few studies identified women’s loss of self or identity: “*(I did not) feel like myself*, *because I felt like that depressed*. *Not even to feel like I have the right to cry freely*’ ([47], p.1567). They mourned the loss of the person they once were and the loss of their old life in their home country: *“Everything needs to change*, *my former life needs to change to a ‘mother life’ now so… it is not an easy something*” ([40], p.759).

#### I feel desperate

Women recognised that something was wrong when they cried without reason and described having little control over their emotions: “*I felt I couldn’t bear such a heavy responsibility*. *I was emotionally very unstable in those days*. *I felt I could not look after her”* ([32], p.601). For some women, their feelings of sadness and desperation were overwhelming and caused them to want to escape the circumstances they were in: “*I felt something different*, *something sad*. *Whenever she started to cry*, *I felt desperate*, *with a desire to run away”* ([11], p.444). One woman in Ornelus’ study described the earliest months of motherhood as the hardest:

*I got very depressed…I even thought about putting him in the laundry basket*. *I didn’t know what to do with him anymore because he cried a lot*. *I couldn’t put up with him anymore and I would start to cry*.

([47], p.1564).

In some studies, a few women reported that they felt like dying: “*Taking my life…*. *Ending it up*” ([40], p.758).

#### They’ll think I am crazy

Many participants came from countries where motherhood affords a high status. This is in part due to the importance placed on children; their role in continuing the family and community [33]. As a consequence, when women experienced feelings of sadness and despair, they were too afraid or embarrassed to say anything [11, 42], believing that as mothers they “*should try to be happy*” ([32], p.606). They were concerned that they would be labelled by family and others as 'crazy' and as unfit mothers [11, 32, 34, 38, 39, 41, 42], “*Because depression*, *like if you see the symptoms of depression*, *it’s a mental illness*. *The minute you say mental illness in my country*, *you are crazy”* ([41], p.166).

The fear of being judged a bad mother was emphasised across accounts [33, 34, 38]. This experience is highly influenced by prescribed gender role expectations in most cultures and is emphasised by women as they make sense of their feelings (see Theme 2). The shame and stigma associated with how these women felt could lead to fears that their baby would be taken away:

My biggest concern is that people will think I’m crazy or that I’m not normal and then they’re going to come to the conclusion that I’m not able to take care of my child and then they’re going to take my child from me.

([32], p299)

Consequently, women ignored their feelings, refusing to talk about it and pushing themselves to carry on their daily duties [11, 32–35, 39, 40, 42]. They believed that if they were to go on, their symptoms would eventually go away as their children grew up [39]. They also found many ways to make sense of or explain this experience.

### Theme 2: Making sense of my feelings

Many women did not view their symptoms as an illness and made sense of it in other ways. Some explained that PND was not recognised in their culture. But for most, the cause was stress-related; stress related to their status as migrant, as a woman and as a person living in poor socio-economic circumstances: “*I think it is about the stress… and the (lack of) community (in the host country)…*.’([40], p.758). Several subthemes emerged in this theme–“You cannot talk about PND”; “Living as a migrant can be difficult and precarious”; “Loss of social support and cultural traditions”; and “Gender roles and the good mother” (see Table 3).

#### You cannot talk about PND

The reviewed studies showed that migrant women rarely recognised or understood the symptoms of PND [11, 38–40]. Some women indicated that they had no knowledge of PND in their own country:

Even though I’m educated I didn’t know that there is something called postpartum depression. I was so suddenly alarmed and scared. Am I turning into an evil person? Am I a bad person?…

*(*[44], *p*.*739)*

These feelings were considered a part of motherhood and the postpartum period; everyone experiences it. One Indian woman in O’Mahony’s study believed that everyone felt that way:

In my culture, they don’t believe in postpartum depression. They say, “Everybody feels it, but they come out of it …” I never got help or explained my problem to any Indian woman because I know how they think … they wouldn’t understand. As a joint family, you tell anyone in or outside your home, people start to talk. “She is feeling this way so maybe she’s going crazy” … slowly the problem gets bigger. That’s why we hide this.

*(*[44], *p*.*739)*

Women were also concerned that their husband would be disappointed with them [32–34], that they may be rejected by their new family, forced to separate from their husbands [11, 32, 34], and that they would bring shame to their families if they were found to have a mental illness [11, 32, 34, 39]. One participant in O’Mahony’s study reported her anxiety about others’ views in the home country: ‘*Back home*, *if someone has this problem*, *everyone gossips*, *you get this feeling that people are not dealing with you normally or as if you are abnormal almost*…” ([46], p.307). A woman in Gardner’s study remarked that stigma attached to mental illness would ‘*follow me around*’ ([40] p.760). Women’s feelings of guilt, embarrassment and humiliation were exacerbated as a result [11, 32, 33, 42].

As a consequence, some women attributed their symptoms to physical causes including physical exhaustion, fibromyalgia, rheumatism and chronic fatigue [39] or physical changes due to childbirth [42]. Women in Ahmed’s study stated: *“You feel depressed because you feel drained of energy*, *because of the childbirth process*…*”(p298)*. Negative body image perceptions and even body odours were also reported, *“I think it’s basically because you reek*…*physically reek (smel)”*, and *“Sometimes you just get upset because you look at yourself and you’re so fat and just a mess*, *and that can lead to some form of depression*.*”* ([42], p298).

#### Life as a migrant is difficult

Participating women almost always reported significant stresses related to moving to a new country including financial difficulties, employment, housing and access to services. This, they said, accounted for their feelings or depressive symptoms. For example, one woman in Gardner’s study stated: ‘*Yeah I know help is at hand*.. *but look at me*! *This house- I don't have landline*. *I have a phone*. *I have no credit on that phone*. *Even if I am in trouble*, *who am I going to call*?” ([40], p.759). This situation was exacerbated by having a precarious migrant status such as being on temporary visas or seeking asylum without legal documentation.

*It's related to our life*, *you know it's very hard to live; especially my husband*, *his visa is not permanent*, *until he get a permanent visa it's hard to us; he's got no job and I am jobless too; it's a bit hard to live like that… when I think a lot about this*, *I feel a bit depressed*.

([35], p. 670)

Because when you’re legal you can take the child to the daycare and look for a job… if you don’t work, it’s like you’re dead, being alive. We want our papers so we can progress; not so we can leave or be a load to anyone, but just to work—to buy a home and give our kids a good life… I get depressed because I can’t live like normal people because I’m always thinking if I leave or if I stay…

([43], p.720).

In this situation, women felt they could not ask their partners to take any time off work to be with them and to help them care for their baby. In the study by Morrow [32], women spoke about their husbands’ long working hours and that they were often alone following birth because their husband had to be at work,

I told him he could request parental leave, but he was worried that the company would fire him, so he didn’t ask for parental leave. So nobody took care of me.

*(*[32], p.604)

#### Loss of social support and cultural traditions

Central in all studies was women’s distress at the loss of family support and limited friendship networks at this most important time in their lives. Isolation from family also meant that many women could not undertake the cultural practices related to pregnancy and the postpartum period that were important to them. Motherhood was experienced or understood as a celebrated time in their culture, a time when the mother is surrounded by family and close friends who help take care of her and her baby [11, 40, 42]. For example, *“(back at home) The families are so big and so supportive*. *We’re always in touch*… *always gathering around someone*.” ([45], p.E51), and “*You are attached to the family house you are not on your own*..” ([40], p.759). For some women, it is traditional to stay with their mothers (or mother in law) after birth [32] and the practice of a 40 day recuperation period is also common [32–34, 38, 42]. During this time, the pressure of taking care of the baby while trying to recover from birth is alleviated by the constant care from family members. In a new country without family, this is not possible. Some participants attributed their symptoms to ailments caused by not following cultural traditions, “*In fact I knew why I caught a fever*. *I was outside on the street and I felt the wind…but then I got sick because of that*.. *"* ([32], p. 602).

With the lack of traditional female supporters, migrant women turn to their husbands: “*I cried for no reason and sometimes I phoned my husband at work just to cry*. *I have no other friends or family to talk to*. *I feel exhausted and depressed*” ([34], p. 69). However, many women also reported that their husbands were unhelpful (see following subtheme).

In contrast, some women believed their distress was exacerbated because of conflict or clashes in cultural beliefs between the woman and her parents in law [33]. Sometimes women were pressured by their in-laws to abandon their cultural practices. These conflicts were heightened because the women did not have their own families around. One Indian woman described her distress at the response of her husband’s family when she stayed with her own mother following birth:

*They said a lot of things to me that were very upsetting*, *like this* (staying with her mother) *was an old tradition*, *they didn’t know why some people were still living in the past*. *After having the baby*, *I spent time at my parents’ home*, *about 13–14 days*. *My in-laws would not phone me or talk with me properly*. *I was very upset at that time*.

*(*[32], *p.608)*

#### Being a ‘good’ mother and wife is culturally expected

For the majority of women in these studies, there was an expectation that they would perform the traditional role of the good mother and good wife. Being a ‘good’ mother was central to women’s identity and many worried that they did not meet expectations. A good mother and a good wife was described as ‘a strong person’ [11] with a duty to 'carry on regardless' [39], to be responsible and in control of their new role [34], and to cope with the demands of caring for children, husbands and households [11]. These feelings were made worse by the mixed and often critical reactions of family, especially parents-in-law with high expectations, and women report feeling unworthy of their family [32–34]. One participant remarked: “*I felt the pressure that I almost wanted to die; I felt the pressure mainly when I had to deal with my in-law*” *(*[32], p.607). There was also pressure to keep it in the family with women being told, “*don’t hang your dirty laundry out*” ([39], p.215). Women born in South American countries reported:

The mothers .. are expected to do everything. And people really think less of them when they have depression. Some women (may) think they cannot take it anymore, and that she is going to explode her emotions

([11], p.445).

In the context of traditionally defined male and female gender roles, women found they were dominated by, and under the control of, their male partners. In O’Mahony’s study [45], some women believed that their distress and sad feelings were directly related to a difficult relationship with their husband. Some women were forbidden to seek paid work or to take language classes, and some experienced an abusive relationship [43]. For many reasons, as noted above (including precarious status as a migrant; financial issues; language barriers), they were dependent on their partner and were unable to leave even if that is what they wanted.

Because we make argument, sometimes he hit me. I was alone and nobody to help me. Sometimes I was very nervous. I felt I’m his slave not his wife. He wanted everything to his hand and make control for everything in my life. I don’t think this is life..

([43], p.720).

### Theme 3: Dealing with my feelings

Without an understanding of PND, women dealt with or came to terms with their symptoms in various ways. There were three sub themes identified in this theme: ***“I just get on with it”; “I am strong”; and “It’s good to talk and feel cared for”***.

#### I just get on with it

It was commonly reported that women felt that their depressive symptoms were all part of being a mother and so they are meant to deal with it by “*praying to Allah*” and “*keeping* (themselves) *happy*” ([38], p.256). Consequently, these women felt that “*there’s no point talking about this problem because everyone has to do it*, *it’s what women need to do*, *they have to do it*” ([38], p.256). Others considered that it would just ‘go away’. Women were reluctant to take prescription medication such as antidepressants, with one woman agreeing with her mother’s advice that that if she “*started taking tablets*, *then this issue* (PND) *would become bigger*” ([32], p.612).

#### I am strong too!

Importantly, women also demonstrated personal strength and resilience. Women spoke about changing their attitudes and expectations to adapt to their situation, engaging in introspection, and modifying their daily routines:

You have to overcome it yourself I think… it’s your own will which helps. I tried changing my routine, I tried to make myself more busy and do more things… like, try to make things easier for myself. Spend a little more time for yourself, and that helps.’

*(*[42]*, p.300)*

O’Mahony [44] emphasised the importance of women’s personal strengths and resolve to work hard to achieve their goals:

I *want only to have a better life*… *My husband used to abuse me because of my bad English*. *I don’t want that to happen again*. *I want to learn about problems and how to solve them*… *I need to take care of myself in order to take care of my baby*. *Having experienced this*, *slowly you get more strong*… *having knowledge*, *you know how to protect yourself and your baby*… *your life gets better*.

(p.740)

Some studies also demonstrated the strength and enjoyment that women gained from their infants [40, 41]:

I can be strong because of him, you know, I don’t cry, I don’t try to kill myself, so he gave me the power. And I think my life is better because even when with the big problems that I have now, he makes me laugh, he try to talk to me, he’s a very happy baby, so I think my life is better because of him.

*(*[41]*, p.167)*

Returning to work was a means for some women to deal with the issue and to make them feel better: “*But the best thing for me is to go out…*. *What I did*, *I went out and I got a salary—I start working and all of a sudden it took my mind off it”* ([40], p.760).

Some women referred specifically to their faith and religion as a resource or as a source of strength in their recovery/dealing with their depressive symptoms: “S*ometimes I get back to my praying and everything and that helps a lot*, *more than what people could help me”* ([35], p.675). For these women, their spirituality was very much a part of their culture and way of life, it was nurturing or part of their health practices [34, 35, 40].

I believe in God, I prayed a lot. Really I am proud of myself … I came all the way from Africa to live here and have family. I keep that successful. So that gives me more strength, I say ‘wow … I did it,” I’m thankful because all the time God was with me to help me. I never feel inferior, every single thing, I counted as success.

*(*[46]*, p.309)*

#### It’s good to talk and feel cared for

Women appreciate having someone to talk to, and someone to ask them how they are feeling/coping. In all studies, the most important source of support was ***informal support*** from family, friends and in some cases from husbands. When available, this support is of substantial help emotionally, socially and sometimes financially [11, 34, 39, 40, 42]. Ahmed et al. [42] report that approximately half of the sample in their study either travelled back to their family or had the women’s mother visit in order to help her with the new baby. These women received help with cooking, household chores and taking care of the baby as well: *“They (mother and grandmother) helped me go on*… *Your own family needs to give you strength*, *to give you support*.*”* ([11], p.445). Additionally, Hispanic women in the USA made use of a ‘compadre system’ where women in the community would support one another [11]. As noted in theme 2, women did not spontaneously mention support from their husbands, but when prompted women did not expect their husbands to provide practical help; any help forthcoming was based on his discretion [38].

Women also found many benefits from ***formal*** community-based support provided by health and other professionals and services. However, these services were often discovered through chance [39, 40, 42, 45], and women reported it distressing when formal services ceased, for example once they had been in the country for a certain length of time or their child started day care [42, 45]. Having a professional bring up the topic of mental health was appreciated by some, leaving women feeling cared for and comfortable to talk about their feelings [42]. Additionally, they felt a sense of support from professionals as a consequence [32]. Genuine care, availability and efficiency of staff were of importance to many women. In addition, some women expressed their appreciation for those that went above and beyond their roles to ease these women’s lives:

They (staff at a francophone settlement support centre) helped me by trying to find places (to live) where it would be least expensive for me, which I appreciated a lot

([42], p.299).

Women also found many benefits from support groups in the community which provided informal respite from chores and childcare in a relaxed and welcoming social setting [34, 39, 42]. This positive attitude towards group support was shared by migrant women in Canada:

I think that the thing that I looked most for (in a community service or support center) was something that allowed me to meet people, just get out of the house and meet others, like new mothers, people that I could speak to… people who are in the same position as myself.

([42], p.300)

Hearing the trials of other women enabled others to put into perspective their own problems. They valued talking to someone about their problem, reflected in a statement made by a Bangladeshi woman: *“If you don’t tell someone about the sadness in your mind*, *no one will realize*, *it will stay inside”* ([38], p.257). However, some women preferred one-to-one support, wanting a more confidential form of help. There were also mixed views on telephone support, some having negative experiences and others finding this a more convenient and anonymous way to receive support [45].

### Theme 4: What I need to change the way I feel

In contrast to the sense of resilience presented in the theme above, all the studies reported that women were often reluctant to seek help for their emotional health [11, 32, 34, 35, 38–42, 45, 47]. As identified in Themes One and Two, women were reluctant to talk to people, particularly health professionals, about their feelings. One woman in Ahmed’s study stated, “*I feel shy telling people about how I feel*, *like I feel embarrassed…*. *It’s kind of humiliating… So I keep it to myself*, *I don’t tell nobody about that*.’ ([42], p.300) Concerns about stigma and being labelled a ‘bad mother’ also contributed to women not seeking support within their own communities.

I didn't just……. Open up totally…… to them.I wouldn't want to…. If one person knows about it, two people know about…..three people knows about it….. so I just cut off. I think it's just,it's just that I don't want the stigma to just keep following me around.”

([40], p.760)

Furthermore, in many cultures it is not acceptable to seek help for mental health problems [11, 44, 48]. In some instances, women cannot afford health care [11, 43]. Women made meaning of their depressive symptoms in many ways and few were unlikely to see their distressing feelings as a medical problem: “*I told her (nurse) about my backache*, *but about feeling depressed*, *I didn't feel comfortable*. *It's my problem; even if they ask me… they can't do very much* ([35], p.674). Similarly, women did not believe it was the role of their doctor (general practitioner—GP) to address emotional problems and that a GPs concern was only physical ailments [32]:

Well we think, I have this (emotional) pain because of my own situation. What can the doctor do about it?….What type of medicine can you get to have peace in your mind?

([38], p.257)

The synthesis of these 13 studies identified two major issues related to accessing health services; first, women want more information about PND and the services that are available and second, services need to respond more appropriately to women from migrant backgrounds.

#### I need more information

Women indicated that prior to birth they did not have enough information about emotional problems or where to go for help. Even those who were aware of PND were taken by surprise, having assumed it was not relevant to them, with one woman explaining *“*..*When the (prenatal class) instructor touched on postpartum depression*, *I just closed my ears and thought ‘that’s not going to be me’*, *they just skim over it… because it is not something that affects so many women…*.*”* ([32], p.612). Migrant women in the UK also reported that even though leaflets were provided, they remained unprepared for the feelings they may have as a mother and there was limited access to their preferred mode of face-to-face education [39]. Hispanic also women asked for more education:

We really need classes (about) the symptoms of depression, and that if we have these feelings we should be encouraged to call someone and not be ashamed, to look for help, but they do not say anything.

([11], p.446)

Poor knowledge about PND among partners and immediate family possibly heightens the stigma associated with depression. Morrow [32] states that this contributes greatly to the lack of support women get from their husbands. Middle Eastern women in Australia suggested that education offered to their close family would be of great benefit to their own lives while living with PND [34]:

I hope that they will include husbands and especially my mother and mother-in-law in this education program so that they will also know about this condition. They will be more understanding and they will not call me crazy or bad mother.

([34], p.70)

Half of Ahmed’s participants claimed to be unaware of any support services available to them and that the main services utilised were local support centres for new immigrants and language centres [42]. Another study in the UK [39] found that women had mixed knowledge about available support services; or, as reported by O’Mahoney, some women had no knowledge of services at all, and that this was exacerbated by language difficulties:

…you don’t know where to go, what to do, who to trust, especially when you are coming by yourself… you believe that you speak English, but when you get here you realize that you don’t.

([44], p.739)

Women were also critical of some services, for example hospital postnatal care. Women were negative about the reduced hospital discharge times pointing out that they “*need rest at that time*, *and they just send you home*” ([38], p.255). One woman complained of not being helped by the staff saying that “*the nurse is there to help but they say ‘you do it yourself’*” ([38], p.255). They were also upset that some services ceased abruptly for example, when the midwives or health visitors stopped coming to their home in the first week or so after birth [40].

#### They don’t understand me

Being able to clearly communicate with a health professional was noted as the most significant barrier to seeking help for depression [38, 39, 42]. In almost all of the studies, the authors report women’s difficulty communicating with health professionals about their feelings. Some women stated that the physical symptoms of depression were so debilitating they had no energy to attempt tackling their language difficulties and try to explain what they felt [39].

The other major barrier reported in most studies was the attitude of staff in health and community services:

I also think it’s really important, the attitude of the people who are working there, healthcare workers who are meant to help you. You can really tell if someone is there just to do their job, or if someone really cares about you and wants to help you, I think that’s the most important thing in what makes me hesitate in using these services.

([42], p.300)

Some women were discouraged from using services when they encountered health professionals who were disinterested or too rushed to listen to them [32, 38] or they had very long wait times [42]. Women reported that they were given incorrect or incomplete information because staff felt that they could not communicate with them, leaving them unsure of the appropriate places and people to talk to [11, 33, 38, 39, 42]. Lack of support from health professionals both in detecting or addressing women’s mental health issues resulted in a relapse in PND symptoms for some women during their next pregnancy [39].

The cultural sensitivity of health professionals towards migrant women was an important factor influencing help seeking. Hispanic women reported feeling “*shuttled from service to service*” because no one knew how to take care of their “culture” ([11], p.445). Jordanian women spoke of being torn between their own cultural practices and Western health advice, having health professionals placing pressure and unrealistic demands upon them to change their beliefs and behaviours [33].

## Discussion

In this meta-ethnographic study migrant women experiencing depressive symptoms described being alone, worried and angry. Women did not identify their symptoms as an illness and believed their feelings were due to life stresses stemming from their status as a migrant, financial difficulties, separation from family and friends, and constraints in their ability to participate in relevant cultural practices related to pregnancy and the postpartum. The fear of being labelled a ‘bad’ mother' meant they did not seek help or accept treatment. Most did not think seeking help or advice from a health professional was appropriate or necessary. When they did seek help, they experienced difficulties communicating with professionals.

In many respects, migrant women’s experience of PND parallels the experience of many non-migrant women. The previous meta-syntheses describe a sense of ‘spiralling downwards’ [18], ‘pervasive despair’ [19] and the progression of PND from ‘crushed maternal role expectations’ to’ going into hiding’; to ‘loss of sense of self’ and experiencing ‘intense feelings of vulnerability’ [20]. Importantly, a few of the studies in this meta-ethnography emphasised migrant women’s strengths and capacity to address their feelings and situation. Women demonstrated resourcefulness in terms of positive thinking and motivation to participate in activities outside the home and emphasised the benefits of having someone to talk to. For some, their faith was important.

**Fig 2:**
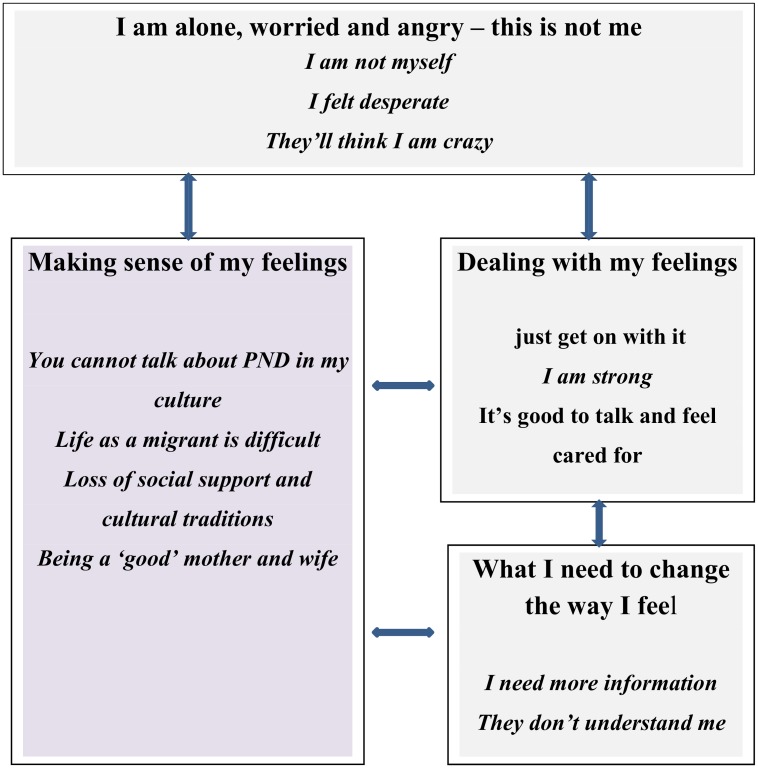
Study themes.

In contrast to the previous syntheses, the focus of most of the 13 studies included in this meta-ethnography was on barriers to help seeking and as a result, we have not been able to identify a trajectory for migrant women from initial recognition of their feelings, through to diagnosis, living with PND and to resolution. Instead, what our meta-ethnography explicates are women’s attempts to make sense of their experience and their responses and actions in relation to their feelings. Our analysis reveals a more complex story of the interrelationship of family, cultural and socio-political factors that both contribute to the development of depressive symptoms and influence the motivation to seek help. Morrow [32] and O’Mahony et al. [43–46] emphasised the importance of studying migrant women’s experience of PND from an intersectional perspective, taking into consideration gender, class, race, culture, and ethnicity. We argue that the theme ‘**Making sense of my feelings**” articulates the multiple factors that challenge migrant women when they are becoming mothers in a new country. Contextual factors (no support from family and community, migration status, socioeconomic status, gender roles) explicated in this theme are risk factors for the development of PND symptoms in explanatory models of PND [49] and are also factors that mediate help seeking behaviours[50, 51]. Furthermore, the theme ‘**What I need to address my feelings**’, alerts us to the limitations of current health services in addressing women’s needs and identifies how this can be remedied. Fig 2 illustrates the relationship between the themes and sub themes identified in this study and we discuss the key issues below.

### The socio-cultural and political context of PND

In their meta-synthesis of non-immigrant women’s experiences of PND, Mollard [20] concluded that women’s narratives reveal the importance of understanding the sociocultural context of birth and PND as embodied. She noted that women most often described their depression in the context of personal relationships and social networks rather than as either a problem with health care or other possible biomedical or psychological factors. This experience is similar yet perhaps magnified for migrant women. In this meta-ethnography, migrant women explained that their PND symptoms were a response to displacement from their family and community; their financial and employment status, their status as a migrant, social isolation and other practical concerns, rather than an illness. O’Mahony and Donnelly [43] used post-colonial feminism to examine how structural inequalities and oppressive forces shape the health and illness experiences of migrant women with PND and their access to appropriate care and treatment. They and others [52] point to difficulties in gaining employment, low paid work resulting in financial difficulties, unequal treatment or discrimination in the workplace and the dependence on their employers experienced by migrant women, as limiting the control that women have over their ….lives and the care of their family. Ultimately discrimination can undermine a woman’s confidence, disempowering her and making it less likely that she will seek support from services, thereby impacting negatively upon her health [43]. These authors report that this experience then impacts upon how women cope, typically viewing their feelings as something they simply have to bear, rather than something to seek help with from a health professional.

Migrant women reported strong feelings of isolation, lack of social support, and the inability to perform traditional cultural practices and this is supported by other research [53] Evagorou and colleagues [52] highlight the increased risk for mental health problems among women from countries and cultures where special emphasis is given to the perinatal period, where the new mother is greatly appreciated and receives significant support from family and society. Without this support, in their new country, women may be more reliant on their intimate partner. This shift in role for men may be difficult for many women and men as traditionally in many cultures it is female relatives that provide this support. If this relationship is not strong or cohesive, relationship problems may contribute to poor mental health in this vulnerable time for women.

The studies in this synthesis and by others [52] suggest some migrant women believe that their situation would be different if they gave birth at home near their families. There, with support from their family, they believed they would have been able to participate in traditional practices. For example, Rodrigues et al. [54] found that “the failure to observe rituals and dietary practices associated with childbirth, such as the use of special diets perceived to be nutritious, and body massages with oil, was associated with the experience of depression” ([54] p.1803). However, there are mixed reports on the impact of participating or not participating in cultural practices related to birth. In a recent systematic review, Fisher et al (2015) noted that postnatal practices did not necessarily reduce the incidence of depression and they reported that the prevalence of PND in LMIC is in fact higher than in HIC. It is possible that, in some circumstances, migrant women have an idealised view of the support they would have received in their home country. Others also report that despite the great physical relief that these practices may provide, these same practices can be a source of conflict [52, 55]. Many women may in fact participate in certain cultural practices to keep the peace with family, in-laws or other community members.

### Stigma and idealised motherhood

The stigma associated with mental health issues, shame and embarrassment about symptoms, fear of being labelled, a lack of awareness about PND and/or a lack of family/community support were palpable in this analysis. Feelings of despair, isolation and desperation were accentuated by societal and cultural views on the nurturing role of the ‘mother’ leaving women struggling with the pressure to be ‘a good mother’, and into hiding their negative feelings because they did not want to be seen as being unable to cope [16]. As a result, there is likely to be low reporting or disclosure of previous or current mental health symptoms [56]. Indeed, some women fear that coming forward with what they are experiencing may put them at risk of losing their infant [13].

In the previous meta-syntheses, non-immigrant women struggled with conflicting expectations of the idealised notions of Western motherhood and the reality [18, 19]. In our meta-ethnography, while migrant women described a contradiction between the expectations and reality of motherhood they explained it differently. Migrant women were less likely to question their status as mothers but appeared distressed that they could not perform their mothering role as they had anticipated due to a lack of traditional supports around them [41]. Similarly, Abrams and Curran’s [57] study of PND and women from low income and minority groups found that women struggled to develop a sense of self as ethical and nurturing mothers whilst also experiencing feelings of guilt, failure, and a compromised sense of maternal competence. Yet the these mothers did not describe the loss of self or autonomy in the same way that literature on White, middle-class mothers has described [57].

### Resilience

Despite the many challenges faced by mothers in these studies, examples of resilience and strength were also described as they adapted to these multiple challenges. In this synthesis help came in the form of a bond with their baby; relationships with partner and family; contact with and assistance from other migrant community members; connecting with a trusted healthcare provider or through employment. Commonly, religion and spirituality provided a source of strength and support to women who otherwise had little to fall back on for support. McLeish [58] and Liamputtong [59] also reported that becoming a mother can have positive effects for migrant women, with the baby representing a new beginning and a health resource. Yet, individual and family strengths or resilience are rarely identified by health professionals in routine assessments, where the emphasis is on identifying problems and risk factors [60].

### Barriers to help seeking

The studies included here identified numerous barriers to help seeking. These included language difficulties; a lack of knowledge about PND and the role and availability of services; spiritual and cultural beliefs and practices; and normalisation of PND symptoms. Additional issues included access issues (e.g. transport); childcare availability; and negative past experiences with healthcare.

However, the majority of these barriers represent individual factors or characteristics of women, their families and culture, appearing to place the onus on women for their actions and feelings. Research often fails to account for service system issues and policy-related barriers. As a consequence, these studies tended to place the responsibility upon women to obtain information about PND as an illness and to gain knowledge of available services. Furthermore, Burr and Chapman [61] and Morrow [32] both challenge the implicit assumptions in Western psychiatric discourse that people will be healthier if they talk openly about their emotions and that when there is internal or interpersonal conflict, it should be resolved in favour of the autonomy of the individual. These assumptions are argued to ignore different cultural conceptualisations of the problem and therefore fail to identify culturally appropriate solutions for women from cultures that emphasise the social and communal or collectivist embedding of the individual.

### Limitations

While we conducted an extensive literature search, it is possible that we did not locate all relevant articles. Limiting our search to papers conducted in English may mean we were not able to include relevant data from other HIC that were not published in English. It also appears that no qualitative studies have been undertaken to examine the experience of women migrating from low-income countries (LIC) to other LIC or to middle-income countries indicating a significant gap in this field. In some of the included studies it was not clear if women recruited to the study were economic or humanitarian migrants or both. Given the impact of pre-migration experiences, this is a significant omission and something that future studies must articulate.

We also note that while the rate of PND is reported as higher amongst migrant women, it is not clear in these studies if all participants were experiencing depression. The EPDS and other tools are screening and not diagnostic tools and few studies mentioned using a diagnostic tool as criteria for study entry. The validated cut off score for probable depression using the EPDS is 13 or greater, whereas some of the studies included women with cut off scores of 10 or greater on the EPDS [40, 42–46]. This suggests that while some women were experiencing distress and needed support, they may not have been depressed. It is also likely that some of these women were experiencing high levels of anxiety but this was not screened for in these studies and anxiety was not identified as an issue through our search. It also may mean that the experiences of asylum seeking women or women with post-traumatic stress disorder may not have agreed to participate or may have been excluded as they were not diagnosed with PND.

We were surprised little was said about the differences between the experiences of economic migrants and humanitarian migrants. The study by O’Mahony and colleagues did set out to address how the social, cultural, political, historical, and economic factors influence immigrant and refugee women’s mental health care experiences as well as women’s personal experiences of depression and help seeking. They included eight women who were identified as refugees and 22 women who were migrants but they did not differentiate the experiences of these women in any of the papers. The authors used Kleinman’s explanatory model recognising that the women’s behaviour and healthcare practices were impacted by cultural factors and that differences in explanatory models of health and illness influenced how they sought help for PPD. But this was not differentiated by migration status. This indicates a significant gap in the literature that requires further work.

### Implications of study findings

Research indicates that health care services show reluctance in responding to the emotional needs of migrant women and maintain their focus on the fetus and pregnancy [13, 16, 62, 63]. Positive previous experiences particularly having a positive relationship with health professionals for social and emotional health problems is a key facilitator of engagement in services [9, 13, 64, 65]. As recommended in almost all studies, health professionals require ongoing training and support in cultural competence [66] as well as reliable access to qualified and culturally appropriate interpreters.

Given the importance of family and community connection for migrant women and the known positive benefits of social support for all women, it is crucial that migrant women be offered emotional and practical support as a preventative strategy early in pregnancy [67]. There is also evidence that peer support models can be effective for women with PND [68, 69], although it is important to bear in mind that migrant women may not want community members to know of their problems. As an important first step, health and other services must develop working relationships with the community [66]. In some countries, maternity services are increasingly employing bicultural workers as a conduit between services and the community and to work directly with women [70, 71]. Given the practical difficulties of accessing services, outreach clinics and home visiting services should also be considered.

Future research should continue to examine differences related to ethnicity, socioeconomic status and related social circumstances at both the micro (individual) and macro (structural and system issues). Further research is also needed on how to support women to develop and build on their strengths and more work is needed to build the evidence base for peer support models for migrant women who experience PND.

## Conclusion

This meta-ethnographic study signals the importance of deconstructing the universality of the PND experience. The knowledge that women who are migrants in general report higher levels of depressive symptoms is important not least because it can severely compromise mother–baby interaction and subsequent attachment relationships. While further research is needed to understand this phenomenon more fully, it is important to ensure that health services are able to recognise the specific socio-cultural, economic and relationship needs of women.

## Supporting information

S1 Table ENTREQ statement to enhance transparency in reporting qualitative evidence synthesis. (DOCX)
